# Evaluation of the DTI-ALPS index in neuromyelitis optica spectrum disorder: a cross-sectional study of its correlation with disease duration and disability

**DOI:** 10.1007/s00234-026-04013-9

**Published:** 2026-05-09

**Authors:** Antonio Carlos Senra Filho, Felipe von Glehn, Fernando Cendes, Clarissa Yasuda, Alfredo Damasceno, André Monteiro Paschoal

**Affiliations:** 1 Institute of Physics Gleb Watagin, State University of Campinas, Campinas, Brazil; 2 Department of Neurology, State University of Campinas, Campinas, Brazil; 3 University of Brasilia, Brasília, Brazil; 4 State University of Campinas, Campinas, Brazil; 5 Department of Neurology, State University of Campinas, Campinas, Brazil; 6 Institute of Physics Gleb Watagin, State University of Campinas, Campinas, Brazil

**Keywords:** DTI-ALPS, Neuromyelitis optica spectrum disorder (NMOSD), Multiple sclerosis (MS), Brain, Diffusion MRI

## Abstract

**Purpose:**

This study evaluates the utility of the Diffusion-Tensor Imaging Analysis along the Perivascular Space (DTI-ALPS) index as an imaging marker for neuromyelitis optica spectrum disorder (NMOSD). We investigated its correlation with disease duration, clinical severity, and its specificity relative to a Multiple Sclerosis (MS) cohort.

**Methods:**

We conducted a cross-sectional study involving patients with NMOSD (*n* = 21), RRMS (*n* = 42), and healthy controls (*n* = 34). DTI-ALPS values were calculated from 3T MRI data using a standardized automated pipeline. Correlations between DTI-ALPS and clinical metrics—including the Expanded Disability Status Scale (EDSS) and disease duration—were analyzed. Comparisons were performed between all three groups to assess the index’s ability to differentiate neuroinflammatory pathologies.

**Results:**

NMOSD and MS patients demonstrated significantly lower DTI-ALPS values compared to controls (P_CON.NMOSD_ < 1.03 × 10^− 7^ and P_CON, MS_ < 4.03 × 10^− 5^, respectively). However, no significant difference was observed between the NMOSD and MS cohorts (P_NMOSD, MS_ = 0,241). In the NMOSD group, a significant negative correlation was found between DTI-ALPS and EDSS (*R* = -0.462, *P* = 0.011), whereas no such association was observed in the MS cohort (*R* = -0.035, *P* = 0.772). Furthermore, disease duration strongly correlated with the ALPS index (*R* = -0.799, *P* = 0.013), with patients exceeding five years of disease showing a more pronounced decline in perivascular diffusivity.

**Conclusion:**

The DTI-ALPS index is a sensitive marker for capturing cumulative neuroinflammatory and neurodegenerative changes in NMOSD. Interestingly, the correlation with clinical disability was unique to the NMOSD group, suggesting that the index may track disease progression differently across demyelinating pathologies. While the index successfully differentiates patients from healthy individuals, the comparable values found in the MS cohort suggest that DTI-ALPS reflects a common pathway of image biomarker for glymphatic or microstructural impairment across demyelinating diseases rather than NMOSD-specific pathology. These findings support its use as a rapid, complementary metric for monitoring disease progression at a group level.

## Introduction

Neuromyelitis Optica Spectrum Disorder (NMOSD) is a rare, severe autoimmune disease that primarily targets six core locations within the central nervous system: the optic nerve, spinal cord, area postrema of the dorsal medulla, brainstems, diencephalon and cerebrum. The disease is characterized by episodes of intense inflammation and demyelination, leading to optic neuritis, often causing significant vision loss, and transverse myelitis, which results in various degrees of paralysis [1, 2]. Unlike multiple sclerosis (MS), NMOSD has distinct pathophysiological markers, most notably the presence of autoantibodies against aquaporin-4 (AQP4), a water channel protein highly expressed in astrocytes [3–5]. These autoantibodies play a pathogenic role, contributing to astrocyte damage, inflammation, and subsequent demyelination. Early and accurate diagnosis of NMOSD is crucial to minimize long-term disability, as timely immunosuppressive therapy can significantly decrease the frequency and severity of relapses [6].

Magnetic Resonance Imaging (MRI) plays a crucial role in diagnosing NMOSD by visualizing abnormalities in the brain, optic nerves, and spinal cord [3, 7]. MRI is particularly valuable in differentiating NMOSD from MS and other neuroinflammatory diseases. In NMOSD, typical MRI findings include longitudinally extensive transverse myelitis (LETM), characterized by spinal cord lesions that span three or more vertebral segments, an unusual feature in MS [8, 9]. Additionally, optic nerve involvement in NMOSD often affects longer segments of the nerve, including the optic chiasm, and is more likely to be bilateral, distinguishing it further from MS. Thus, MRI not only enhances clinical diagnosis but also plays a vital role in monitoring disease progression and assessing the efficacy of treatments.

A particularly useful MRI imaging technique is the Diffusion Tensor Imaging (DTI), which emerged as a valuable tool for studying microstructural changes in the brain and spinal cord in patients with NMOSD (pwNMOSD) [10, 11]. DTI can assess white matter integrity by measuring the diffusion of water molecules along axonal fibers [10]. This imaging technique provides several quantitative measures, e.g. fractional anisotropy (FA) and mean diffusivity (MD), which can assist the study of the organization and density of white matter tracts [12]. In NMOSD, DTI is used to detect subtle alterations in areas that may appear normal on conventional MRI, thus enhancing the ability to diagnose and monitor the disease at an earlier stage.

The DTI-ALPS (DTI–Analysis along the Perivascular Space) index [13] is an emerging metric that has been primarily used as an indirect way of assessing glymphatic system function, which plays a role in clearing metabolic waste from the brain [13, 14]. Recent studies have explored the potential of using the DTI-ALPS index to help identify and monitor neuroinflammatory conditions, [4, 14, 15] presenting a possible application to NMOSD. Given that AQP4 water channels are the primary facilitators of glymphatic flow, their disruption in NMOSD may lead to “glymphatic failure”—a pathological state increasingly recognized as a final common pathway for cumulative tissue injury and neurodegeneration in several CNS disorders [16]. The glymphatic system, which facilitates waste clearance along perivascular spaces, can be impaired by neuroinflammation. As neuroinflammation is a key pathological feature of NMOSD, exploring glymphatic-associated metrics is of high interest [17–19]. Therefore, the DTI-ALPS index could provide valuable information on the involvement of the glymphatic pathways in NMOSD pathology and also give a general status of the white matter integrity.

The DTI-ALPS index is derived by evaluating the diffusivity along the direction of the medullary veins, which run perpendicular to the main white matter tracts at the level of the lateral ventricle bodies. In this specific neuroanatomical region, the projection fibers (running in the superior-inferior direction, or y-axis) and association fibers (running in the anterior-posterior direction, or z-axis) are situated such that the perivascular spaces of the medullary veins are oriented along the x-axis (right-left) [13]. By calculating the ratio of diffusivity in the x-axis relative to the diffusivity in the y- and z-axes within these fiber systems, the ALPS index provides a non-invasive proxy for the ease of water movement along these perivascular channels using conventional DTI data.

This study evaluates the DTI-ALPS index as a promising metric for assessing NMOSD status. By comparing NMOSD patients with both healthy controls and a cohort of patients with Multiple Sclerosis (MS), we aim to determine the index’s sensitivity to clinical disability and its potential as a complementary tool for monitoring disease progression across different neuroinflammatory backgrounds. It is important to note that the application proposed here is to assist other MRI imaging techniques, adding more possibilities to the usual DTI imaging information that is already an ally to neurodegenerative assessment.

## Materials and methods

### Neuromyelitis optica spectrum disorder (NMOSD) patients

This cross-sectional study included 21 consecutively recruited male and female patients diagnosed according to 2015 International Panel criteria [9]. Among them, 15 were diagnosed with NMO, 4 were AQP4ab-seropositive with LETM, and 2 had AQP4ab-seropositive relapsing ON (rON). Additionally, 34 healthy controls, matched for sex and age, were included. In the NMOSD group, 13 NMOSD patients tested AQP4ab-seropositive, while 2 were seronegative. LETM was characterized by acute myelitis with MRI evidence of spinal cord lesions spanning three or more vertebral segments, while rON was defined by at least two episodes of clinical optic neuritis (ON), separated by at least 30 days, without brain lesions outside the optic nerves. The demographic and baseline clinical data of this cohort were previously reported in a study investigating retinal nerve fiber layer thinning [20]. However, the DTI-ALPS analysis presented here constitutes a distinct investigation using an independent imaging processing pipeline. No diffusion-based metrics or statistical correlations involving the DTI-ALPS index from this cohort have been previously published, and the scientific questions addressed in this manuscript do not overlap with prior reports.

Patients were stratified based on disease duration into two groups: those with a short duration (≤ 5 years) and those with a longer duration (> 5 years). This 5-year threshold was selected based on clinical evidence suggesting that the most significant accrual of neurological disability and the transition from early inflammatory relapses to a more chronic, disseminated phase in NMOSD typically occur within the first five years of the disease [21, 22]. This stratification allows for the evaluation of the DTI-ALPS index across distinct clinical stages of the disease spectrum. These patients were recruited during their routine follow-up appointments at the neurological outpatient clinic of the University of Campinas (UNICAMP) Hospital, São Paulo, Brazil, between January 2011 and October 2012. The study was approved by the UNICAMP Ethics Committees for Research, and written informed consent was obtained from all participants. All patients tested negative for anti-HIV and anti-HTLV1/2 antibodies, and other differential diagnoses were excluded. At the time of MRI acquisition, all patients followed a standardized immunosuppressive protocol consisting of an initial combined therapy (Prednisone and Azathioprine), followed by a gradual tapering of Prednisone over six months. Consequently, all patients with more than six months of treatment (*n* = 18; 85.7% of the cohort) were receiving Azathioprine monotherapy at the time of the scan. The remaining three patients were in the final stages of the corticosteroid tapering phase. No patients were experiencing a clinical relapse or receiving methylprednisolone pulse therapy during the study period. Disease severity and neurological impairment were quantified using the Expanded Disability Status Scale (EDSS) [23]. The EDSS is a standardized metric that integrates two components: (i) Functional System (FS) scores, derived from neurological examination across eight domains (pyramidal, cerebellar, brainstem, sensory, visual, bowel/bladder, cerebral/mental, and other functions), and (ii) an assessment of ambulation and global disability. Scores range from 0 (normal neurological examination) to 10 (death due to the disease) in 0.5-point increments, providing a reproducible and sensitive metric for monitoring disease progression.

### AQP4ab testing

All peripheral blood samples were tested for AQP4 antibodies using a commercial, standardized cell-based immunofluorescence assay that utilized recombinant full-length human AQP4 (Euroimmun AG, Luebeck, Germany) [24]. These tests were conducted at the Neuroimmunology Laboratory of UNICAMP.

### Multiple sclerosis (MS) patients

To investigate the specificity of the DTI-ALPS index in differentiating NMOSD from other inflammatory demyelinating diseases of the Central Nervous System (CNS), an additional cohort of patients with Multiple Sclerosis (MS) was included. This dataset consisted of 42 patients diagnosed with Relapsing-Remitting MS (RRMS) according to the McDonald criteria [25]. To ensure consistency in imaging acquisition and environmental variables, the MS dataset was obtained from the same neuroimaging center using the same MRI protocol as the NMOSD and control groups.

### MRI imaging protocol

MRI scans were performed on all patients (NMOSD and MS) and controls using a Phillips Achieva 3-T scanner at the UNICAMP hospital. While the complete clinical protocol included T1- and T2-weighted sequences, only the DTI data were available for this analysis due to ethical committee data-sharing policies. To ensure data integrity, the B0 and Fractional Anisotropy (FA) maps were visually inspected for every subject to confirm that no macroscopic lesions or significant structural abnormalities were present within the specific Regions of Interest (ROIs) used for the DTI-ALPS calculation. This ensured that our measurements reflected the normal-appearing white matter microenvironment. The MRI imaging parameters were: non-collinear echo-planar sequence, TR/TE = 8500/61 ms, flip angle = 90°, SENSE reduction factor equals to 2.5, 32 gradient directions, b1-value = 1000 s/mm^2^, single b = 0 s/mm^2^, 2.0 mm^3^ isotropic voxel size. The diffusion protocol followed the established clinical standards at the time of data acquisition (2011–2012). While these parameters provide sufficient signal-to-noise ratio for reliable tensor estimation in clinical cohorts, they are recognized as a potential factor in the variability of the derived metrics compared to modern high-angular resolution protocols.

DTI preprocessing included correction for eddy currents and subject motion using 3D Slicer [26, 27] (version 5.9) and FSL Eddy [28, 29] (version 6.0.6), skull-stripping, and tensor fitting using a linear least-squares estimator implemented in 3D Slicer. The B0 image was used for susceptibility inspection, and volumes with severe motion artifacts were excluded based on a quality assurance (QA) threshold of 10% of the total acquired volumes. No more than three diffusion-weighted volumes were removed per subject, ensuring that the angular resolution remained sufficient for robust tensor estimation. Consequently, no subjects were excluded from the study based on this criterion, and the integrity of the directional data was maintained for all participants. For the regions of interest (ROI) definition used in the DTI-ALPS calculation, [13] we applied a normalization procedure to assist the WM region location using the MNI brain template 1 mm isotropic FA map, also known as JHU-FA brain template [6]. Bilateral ROIs were automatically defined and to ensure anatomical accuracy, all automated placements were visually inspected on color-coded FA maps by an experienced specialist to confirm correct positioning and the absence of lesions. The final DTI-ALPS index represents the mean of these bilateral measurements. The normalization step was made using the ANTs normalization tool [30, 31] (version 2.6.2-g52bc0ab), which applied a two-step image registration with affine and b-spline methods, where the B0 volume was used to adjust the mutual information optimization function [30] with T1-w MRI template. After the normalization transformations had been performed, the Projection and Association ROIs were placed as described in the Taoka et al. [13] proposition, considering a maximum ROI size of 5 mm^3^.

### DTI-ALPS formalism

The DTI-ALPS calculation was performed using the equation defined in Eq. 1, considering the same procedure adopted by [13].

1 $$\:DT{I}_{ALPS}\:=\:\frac{mean({D}_{xx-proj},\:{D}_{xx-assoc})}{mean({D}_{yy-proj},\:{D}_{zz-assoc})}$$

Where the projection fibers ($\:{D}_{xx-proj}$) and association fibers ($\:{D}_{xx-assoc}$) are placed on the x-axis and the projection fibers ($\:{D}_{yy-proj}$) on the y-axis and the association fibers ($\:{D}_{zz-assoc}$) on the z-axis. All the spatial orientations are defined in the DTI scale, i.e., using the tensorial representation.

**Fig. 1:**
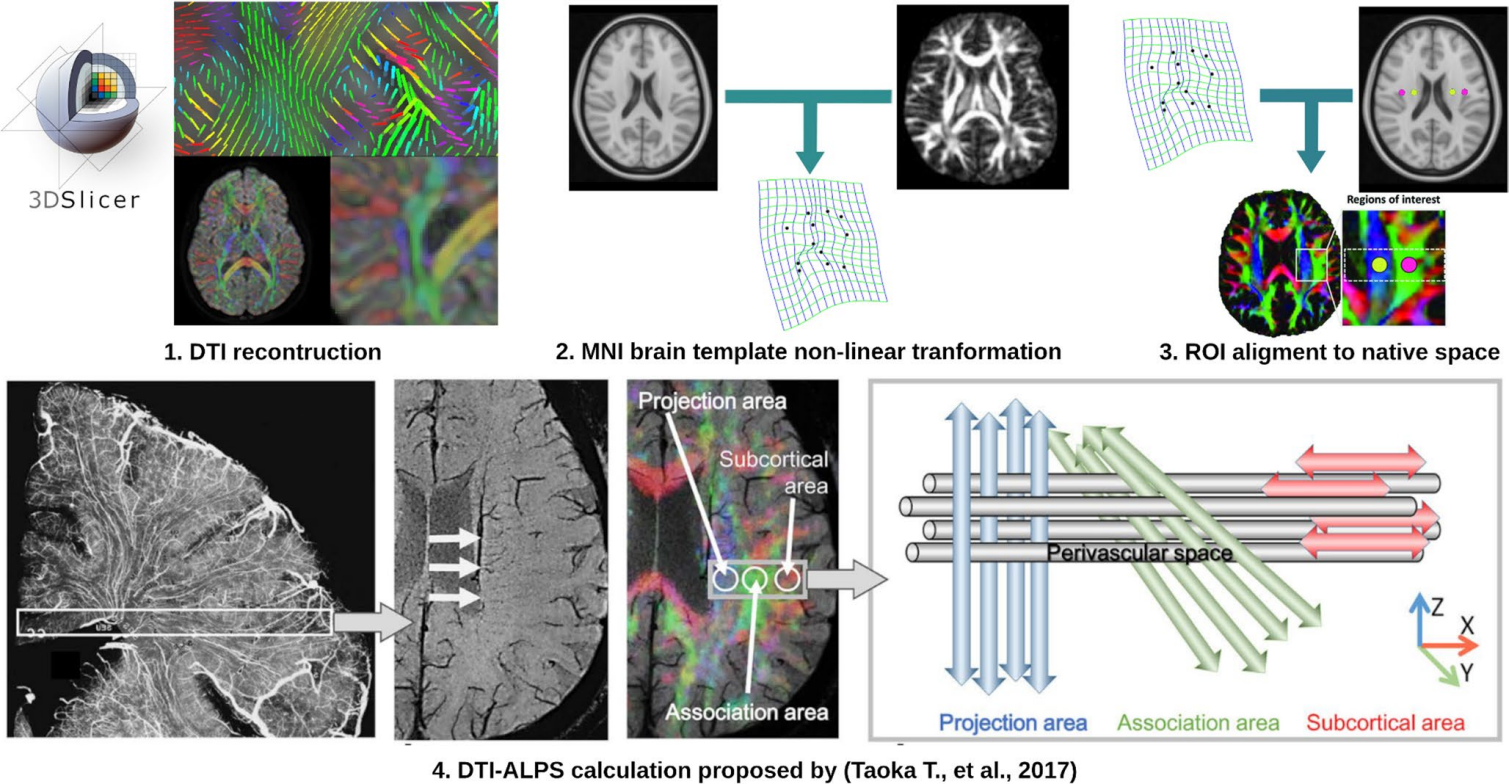
Workflow for DTI-ALPS index calculation, adapted from [13]. The process consists of four main post-processing steps - Step 1 – DTI reconstruction: Diffusion tensor imaging (DTI) data acquired in the patient’s native space are processed to reconstruct the diffusion tensor. This generates maps such as fractional anisotropy (FA) and color-coded directionality maps, which visualize principal diffusion directions in white matter tracts. Step 2 – Non-linear registration to MNI space: The patient’s structural image is non-linearly registered to the Montreal Neurological Institute (MNI) brain template. This transformation allows alignment to a standardized brain space, enabling reproducible and anatomically consistent placement of regions of interest (ROIs) across subjects. Step 3 – ROI alignment to native space: Standardized ROIs, defined in MNI space according to anatomical landmarks around the perivascular spaces of the medullary veins, are transformed back into the patient’s native DTI space using the inverse of the registration step. This ensures that ROI placement is anatomically accurate for the individual, preserving the subject’s native geometry for precise DTI metric extraction. Step 4 – DTI-ALPS index calculation: The final step involves measuring diffusivity along axes perpendicular and parallel to the perivascular spaces in projection, association, and subcortical fiber regions. The ALPS index is then calculated according to Eq. (1) in the Methods section, providing a quantitative measure of diffusivity along perivascular spaces that reflects glymphatic function. Finally, the resulting global DTI-ALPS index is derived from the averaged ROI-based diffusivity measurements in the patient’s native space, following the methodology proposed by Taoka et al. [13]

For the Projection and Association ROI definition, we adopted a spatial localization pattern using the MNI brain atlas [32]. Hence, the WM localization for those regions is more regulated and less influenced by manual definition. Figure 1 represents the DTI-ALPS calculation procedure adopted in the study.

### Statistical analysis

All statistical analyses were performed in Origin (Version Number 2022, OriginLab Corporation, Northampton, MA, USA). Data are presented as mean ± standard deviation for normally distributed variables or median (IQR) for non-normal data. Normality was assessed with the Shapiro–Wilk test and homogeneity of variance with Levene’s test. For group comparisons, we applied parametric between-group comparisons using the unpaired (independent) t-test. When the normality test was not applicable, the Mann–Whitney U test non-parametric test was used. Associations between DTI-ALPS and clinical measures (EDSS, disease duration) were assessed with Spearman’s rank correlation coefficient. Due to the rare nature of NMOSD and the exploratory focus of this study, no formal correction for multiple comparisons was applied. Consequently, p-values should be interpreted as indicative of the strength of evidence in this pilot cohort rather than as definitive confirmatory findings. All tests were two-tailed, and a p-value < 0.05 was considered statistically significant. Exact p-values are reported in the Results. No formal a priori power calculation was performed due to the limited availability of NMOSD cases; this study is exploratory. Results should therefore be interpreted with caution pending larger, prospective validation studies.

### Software sharing information

The DTI-ALPS index was implemented using a public open source strategy, based on Python scripting language. A 3D Slicer^1^ extension is freely available throughout the scientific community^2^. All the Python implementations were aimed at assisting the research and medical community in analyzing and using the DTI-ALPS index in other applications.

## Results

Table 1 presents the demographic, clinical, and serological characteristics of the patients. Patients with a longer disease duration experienced a higher number of relapses, and their EDSS scores were generally worse. Similar to findings from previous studies, [21, 22] only a limited number of patients exhibited cerebrospinal fluid (CSF)-restricted IgG oligoclonal bands, with no significant differences related to AQP4ab serostatus or disease duration (see Table 1).

**Table 1:** Demographic and baseline clinical characteristics are organized according to clinical presentation and disease duration

No. of subjects	Controls	NMOSD	MS	*p* value†	≤ 5 years	> 5 years	*p* value†
	34	21	42		12	9	
Age(years)*	42 (14–76)	38 (14–63)	42 (32–59)	0.5462	39(14–62)	38(17–63)	0.9856
GenderF/M	28/6	19/2	34/8	0.7	10/2	9/0	1
Time from first symptoms (years)*	N/A	5 (0.9–19)	8 (1–24)	N/A	2 (0.9–5)	8.5 (6–19)	< 0.0001
Numberofrelapses*	N/A	3 (1–15)	4 (1–11)	N/A	3 (2–6)	10 (3–15)	0.005
EDSS*	N/A	4 (1–8.5)	N/A	N/A	3.5 (1.5–8.5)	5.5 (3–8)	0.1537
AQP4ab (%)	N/A	19/21 (90%)	N/A	N/A	11/12(92%)	8/9 (89%)	1
CSF oligoclonal bands ^‡^(%) ^**^	N/A	8/21 (38%)	N/A	N/A	4/12 (33%)	4/9 (44%)	0,67

* Median (range)

** Mean ± (SD)

† Mann-Whitney test; two-tailed p value

‡ CSF Oligoclonal bands = two or more cerebrospinal fluid-restricted IgG oligoclonal bands

^a^ Population-based normal range [28]

*P* values are strictly referred to NMOSD group

*AQP4ab* seropositivity for anti-AQP4 antibody, *EDSS* Expanded Disability Status Scale, *NMOSD* neuromyelitis optica spectrum disorders, *MS*MS multiple sclerosis

**Fig. 2:**
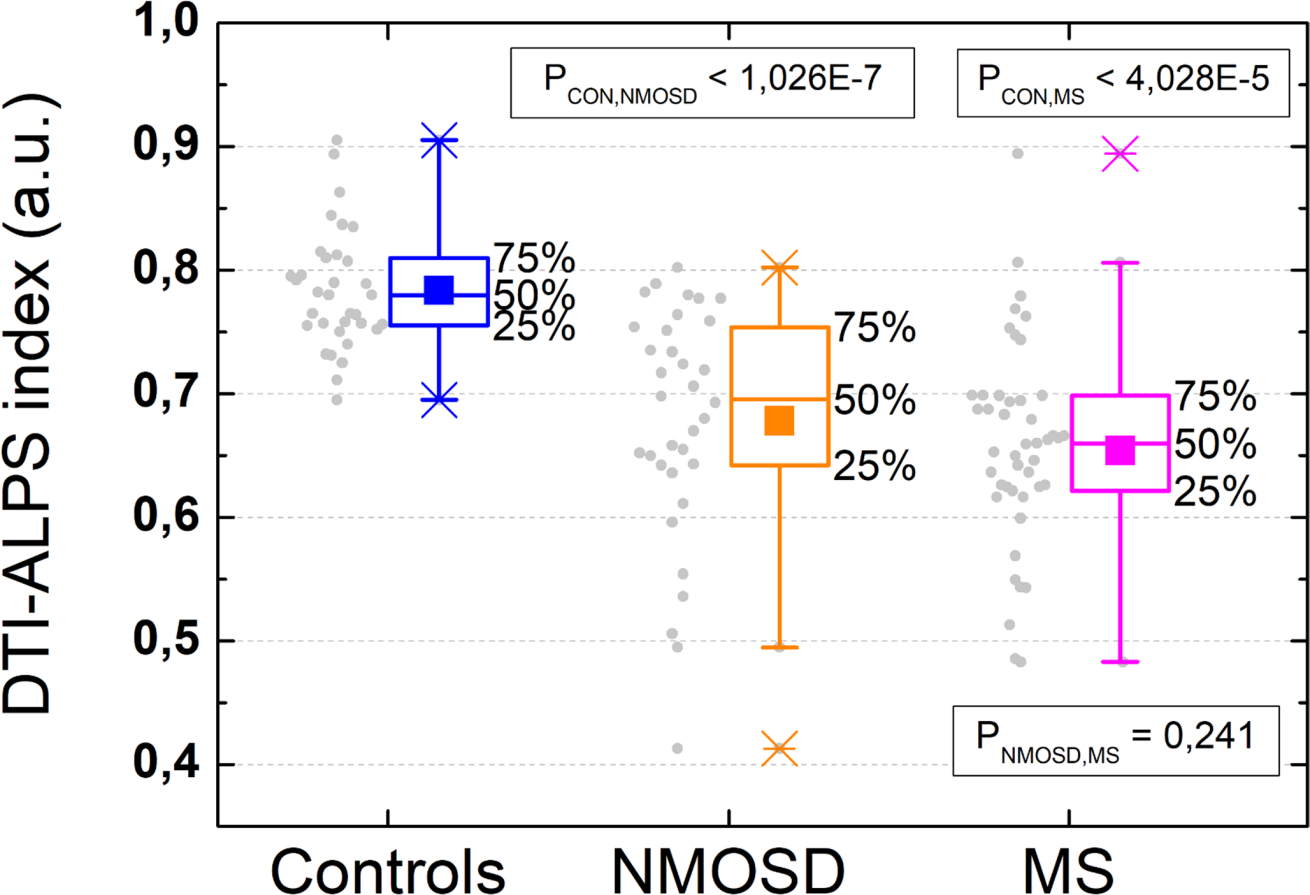
Comparative analysis of the DTI-ALPS index across healthy controls and patient groups. The distribution of the DTI-ALPS index is shown for healthy controls (blue), NMOSD patients (orange), and MS patients (magenta), with individual subject data points overlaid as a jitter plot (gray dots). Statistical comparisons reveal a significant reduction in the DTI-ALPS index for both NMOSD and MS cohorts relative to the control group (P_CON, NMOSD_ < 1.026 × 10^− 7^ and P_CON, MS_ < 4.028 × 10^− 5^, respectively). Notably, no significant difference in the ALPS metric was observed between the two patient populations (P_NMOSD, MS_ = 0.241)

A group-level analysis was conducted to compare the DTI-ALPS index across healthy controls, NMOSD patients, and MS patients (Fig. 2). The analysis revealed a highly significant difference between the control group and both patient cohorts. Specifically, NMOSD patients exhibited significantly lower DTI-ALPS values compared to healthy controls (P_CON.NMOSD_ < 1,03 × 10^− 7^), a finding mirrored in the MS group (P_CON, MS_ < 4,03 × 10^− 5^). However, no significant difference was observed between the NMOSD and MS cohorts (P_NMOSD, MS_ = 0,241).

**Fig. 3:**
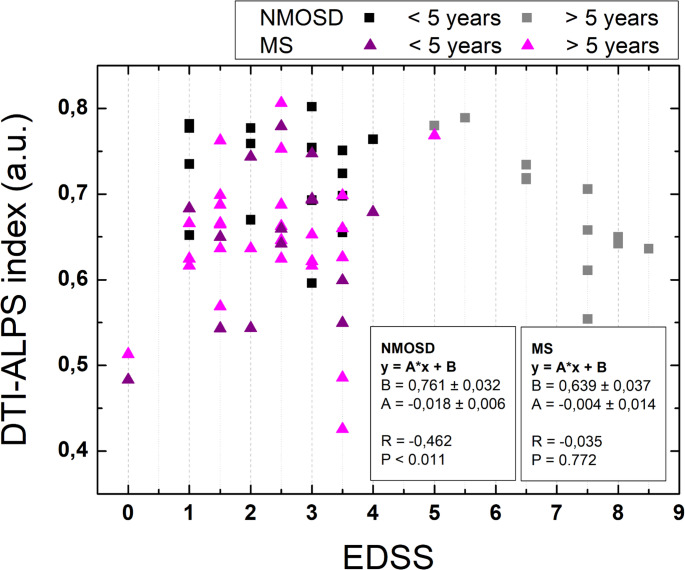
Correlation between clinical disability (EDSS) and the DTI-ALPS index. The scatter plot shows a significant negative correlation in the NMOSD cohort (black and gray squares; *R* = −0.462, *p* = 0.011). Conversely, the MS cohort (purple and magenta triangles) exhibits no significant correlation (*R* = −0.035, *p* = 0.772). Data are stratified by disease duration (< 5 years and > 5 years), highlighting the cluster of NMOSD patients with long duration and high EDSS having the lowest ALPS values. Linear regression statistics for both groups are provided in the inset boxes

Further investigation into the relationship between DTI-ALPS values and disease severity (EDSS) revealed divergent patterns between the pathologies. A notable negative correlation was confirmed within the NMOSD cohort (*R* = −0.462, *p* = 0.011), as illustrated in Fig. 3, suggesting that DTI-ALPS decline mirrors cumulative neurodegeneration in this group. In contrast, the MS cohort demonstrated a negligible association between the ALPS index and EDSS (*R* = −0.035, *p* = 0.772), indicating that this specific imaging marker tracks with different disability drivers in NMOSD versus RRMS.

**Fig. 4:**
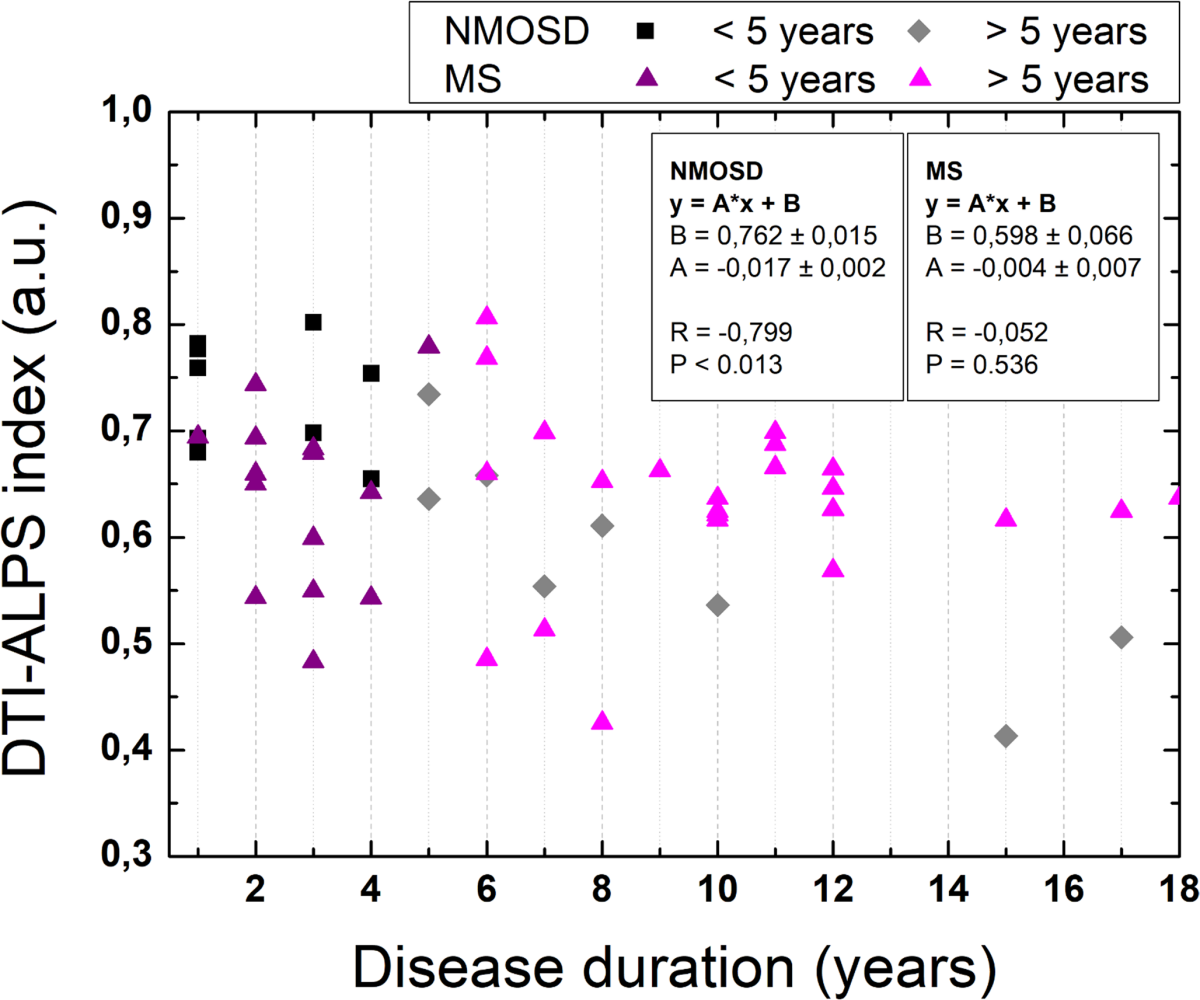
Relationship between disease duration and the DTI-ALPS index. A negative correlation was observed in the NMOSD group (black squares and gray diamonds; *R* = −0.799, *P* = 0.013). In the MS cohort (purple and magenta triangles), no significant statistical association was found between the ALPS metric and disease duration (*R* = −0.052, *P* = 0.536). Regression parameters for both groups are provided in the inset boxes

Moreover, disease duration appeared to play a critical role in influencing DTI-ALPS values. Patients with a disease duration exceeding five years demonstrated a marked reduction in DTI-ALPS, with a significant negative correlation (*R* = −0.799, *p* = 0.013) between disease duration and the DTI-ALPS index, as illustrated in Fig. 4.

Regarding disease duration, a negative correlation with the DTI-ALPS index was found in the NMOSD cohort (*R* = −0.799, *P* = 0.013; Fig. 4). Within this group, lower index values were generally noted in patients with longer disease duration. In contrast, the MS cohort did not show a significant association between the ALPS metric and the time from first symptoms (*R* = −0.052, *P* = 0.536). This observation is consistent with the results for clinical disability, where the ALPS index appears to relate to disease chronicity in NMOSD but does not show a similar temporal relationship in the MS group.

## Discussion

We found that DTI-ALPS values were significantly reduced in patients with NMOSD compared with healthy controls, and that lower DTI-ALPS correlated negatively with both clinical disability (EDSS) and disease duration—most markedly in patients with > 5 years of disease. These results indicate that the DTI-ALPS index captures macro-scale changes in diffusion behavior that track with NMOSD severity and chronicity at the group level.

Notably, our comparative analysis revealed no significant difference in DTI-ALPS values between the NMOSD and MS cohorts (P_NMOSD, MS_ = 0.241). This finding suggests that while the ALPS index is sensitive to neuroinflammatory pathology, it may lack the clinical specificity required to differentiate between these two distinct demyelinating entities. The observed reduction in both groups likely reflects a common pathway of white matter degradation and perivascular space dysfunction. In MS, chronic inflammation and the formation of perivenular lesions (Dawson’s fingers) [25, 33] are known to disrupt the local microenvironment and interstitial fluid dynamics. Similarly, in NMOSD, AQP4-targeted astrocytopathy leads to endfeet injury and impaired water homeostasis [34].

Despite the similar index values between groups, a negative correlation with disability (EDSS) and disease duration was only observed in the NMOSD cohort (Figs. 3 and 4). The reasons for the lack of correlation in the MS cohort remain currently inconclusive [35–37]. It is possible that in MS, the DTI-ALPS index reflects a more generalized and non-specific white matter microstructural alteration that does not strictly track with clinical progression in the same manner as NMOSD. Conversely, the association in NMOSD might be related to the specific AQP4-targeted astrocytopathy and its impact on perivascular drainage, though this remains speculative.

These results suggest that while the ALPS index is reduced in both patient groups relative to controls, its relationship with disease progression appears more evident within the NMOSD spectrum. The sensitivity of the ALPS approach may vary depending on the underlying pathological processes of each disease, and further research is needed to clarify why the index diverges in its relationship with disability across different neuroinflammatory conditions. Therefore, the DTI-ALPS index may serve as a global “sensor” of neuroinflammatory burden and perivascular environment status rather than a diagnostic tool for identifying a specific disease etiology.

Mathematically, the DTI-ALPS index is derived from the ratio of diffusivity along the x-axis (parallel to the medullary veins) to the diffusivity in the y- and z-axes (perpendicular to the main fiber tracts). By focusing on the region where projection and association fibers are orthogonal, the index effectively isolates the directional diffusivity of water molecules that are not restricted by major axonal membranes [13, 15]. Consequently, the information retrieved on this basis serves as a macro-scale proxy for the efficiency of the interstitial fluid (ISF) drainage and perivascular space patency. In the context of NMOSD, a lower index suggests that this directional “path of least resistance” is compromised, likely due to the astrocytic damage and subsequent glymphatic congestion characteristic of the disease.

Because the ALPS index is designed to probe diffusion along perivascular spaces, the lower values observed in NMOSD likely reflect alterations in the microstructural environment around cerebral small vessels and/or more global white-matter integrity changes that affect directional diffusivity [21, 22, 38]. In NMOSD, astrocytopathy mediated by anti-AQP4 antibodies leads to injury of astrocytic endfeet and disruption of water homeostasis [24, 39]; such alterations provide a plausible mechanistic link between disease pathology and a reduced ALPS index. In addition, the finding that DTI-ALPS correlates with EDSS and disease duration suggests that the index is sensitive to cumulative tissue injury (inflammation, demyelination, gliosis, and axonal loss) that can happens after multiple relapses over long disease duration rather than to a single acute lesion burden. It is important to acknowledge that the DTI-ALPS index is an indirect, diffusion-based metric. While it was originally proposed to reflect glymphatic function, the lower values observed in our NMOSD cohort could also be influenced by global, non-specific white matter microstructural alterations rather than isolated perivascular impairment. Given that NMOSD involves widespread astrocytopathy and axonal loss, the reduction in the ALPS index likely represents a composite of both glymphatic congestion and general tissue degradation. Therefore, our interpretations regarding glymphatic dysfunction remain speculative, and the DTI-ALPS index should be considered an exploratory imaging marker of the perivascular microenvironment.

Our results are consistent with the notion that DTI metrics detect microstructural abnormalities in NMOSD even in regions that appear normal on conventional MRI. Previous DTI studies in NMOSD have reported reduced FA and increased MD in optic pathways, spinal cord and selected brain areas [10]; the present study complements those reports by showing that an ALPS-based measure—intended to probe perivascular/glymphatic–related diffusion—also differs between patients and controls and relates to clinical status. The association between longer disease duration and lower ALPS parallels reports that chronicity in neuroinflammatory disorders is accompanied by progressive microstructural degradation. Importantly, however, the ALPS index provides a global, orientation-based summary that is distinct from tract-specific FA/MD measures and may therefore capture aspects of tissue change relevant to brain fluid dynamics and perivascular microenvironment.

Key strengths of this study include the use of an established, reproducible ROI placement workflow (normalization to MNI with inverse mapping to native DTI space) and use of an objective, previously published ALPS formula. The observed group differences and correlations with clinically meaningful measures (EDSS, disease duration) indicate that DTI-ALPS can serve as a sensitive group-level imaging marker for NMOSD-related tissue alteration. Practically, ALPS is relatively simple to compute from a standard DTI acquisition and could be integrated into multimodal MRI protocols as a complementary metric that reflects aspects of perivascular/white-matter integrity not captured by lesion counts or conventional volumetry.

However, the application of the DTI-ALPS index in NMOSD diagnosis is still in its early stages, and several limitations must be addressed. One challenge is the variability in DTI acquisition protocols, which can affect the reliability of DTI-ALPS measurements. Additionally, while the DTI-ALPS index may reflect an indirect measure of glymphatic dysfunction, its specificity for NMOSD remains unclear, as similar changes could be observed in other neurological diseases. More research is needed to validate the use of the DTI-ALPS index in larger cohorts of NMOSD patients and to standardize imaging techniques across clinical settings. The cross-sectional design precludes inferences about causality or temporal dynamics; longitudinal data are needed to determine whether ALPS declines precede, coincide with, or follow clinical worsening. Also, the sample size is modest (21 NMOSD patients) and heterogeneous concerning clinical presentation and serostatus; the cohort’s limited size increases susceptibility to sampling variability and limits subgroup analyses (for example, by AQP4-status or treatment). Another important point to highlight is that all patients were receiving immunosuppressive therapy at the time of scanning; medication effects on diffusion metrics cannot be excluded. In addition, the absence of a disease-control group (for example, MS) prevents any direct claims about the specificity of ALPS changes to NMOSD. The modest sample size (*n* = 21) and the clinical heterogeneity of the NMOSD cohort—comprising NMO, LETM, and rON phenotypes—represent a limitation. However, these clinical presentations are now recognized as manifestations of the same underlying disease process, primarily driven by AQP4-IgG–mediated astrocyte injury [9]. Phenotypes like LETM and rON often represent early relapses, while the classical NMO phenotype reflects a more disseminated clinical expression within the same spectrum. While this inclusion provides a representative view of the NMOSD spectrum, the small sample size may exacerbate correlation coefficients, such as the strong relationship observed between disease duration and DTI-ALPS (*R* = −0.799). Future studies with larger, more homogeneous cohorts are necessary to validate these exploratory findings and minimize the potential for inflated statistical effects. Furthermore, the pharmacological profile of Azathioprine supports the interpretation that our findings reflect disease-related pathology rather than medication effects. Azathioprine and its active metabolite, 6-mercaptopurine (6-MP), are poorly lipid-soluble and exhibit limited penetration across an intact blood-brain barrier [33]. Their therapeutic efficacy in NMOSD is primarily attributed to peripheral immunosuppression—reducing the systemic autoimmune drive—rather than direct action within the central nervous system microenvironment. Consequently, it is unlikely that Azathioprine therapy would directly interfere with or “mask” the water diffusivity patterns captured by the DTI-ALPS index. This suggests that the observed reduction in this exploratory imaging marker is a robust reflection of the underlying astrocytopathy and perivascular damage characteristic of NMOSD [40].

There is also considerable variability observed in DTI-ALPS values among individual patients, as shown in Fig. 2. While this variability supports the use of DTI-ALPS as a reliable metric for group-level comparisons, it raises concerns regarding its application for individual patient characterization. Further research is needed to determine whether DTI-ALPS can be refined or complemented with additional metrics to enhance its precision for individual assessments. Finally, the DTI-ALPS index provides a global assessment of brain fluid dynamics and white matter integrity. However, it lacks the specificity needed to pinpoint changes in distinct brain regions. While these factors necessitate a cautious interpretation of individual scores, our results robustly demonstrate the utility of DTI-ALPS as a group-level imaging marker. Future longitudinal studies with larger, multi-center cohorts will be instrumental in further refining the clinical specificity of the index and establishing its role in personalized patient management. It is important to emphasize that while we observed significant correlations between the DTI-ALPS index and both EDSS and disease duration, the cross-sectional design of this study does not allow for the establishment of temporal or causal relationships. Our findings demonstrate an association between lower ALPS values and greater clinical disability at a single time point, but we cannot definitively conclude that the ALPS index “declines” as the disease progresses within an individual. These correlations may reflect the cumulative burden of past neuroinflammatory episodes rather than a continuous physiological “progression.” Future longitudinal studies, tracking the same patients over multiple years, are required to confirm whether the DTI-ALPS index can serve as a predictive marker for clinical worsening.

## Conclusion

In conclusion, this study demonstrates that the DTI-ALPS index is a sensitive and clinically relevant exploratory marker for assessing perivascular microenvironment alterations in NMOSD. The significant correlations observed with both EDSS and disease duration in NMOSD—contrasting with the lack of correlation found in the MS cohort—highlight the potential of this metric to track the cumulative impact of NMOSD-specific pathology. While the reasons for this divergent clinical correlation between NMOSD and MS remain inconclusive, our results support the use of DTI-ALPS as a valuable group-level tool. Future longitudinal studies are required to confirm if these findings reflect a specific link between glymphatic function and clinical worsening in NMOSD.

## Data Availability

No datasets were generated or analysed during the current study.

## Footnotes

1 https://www.slicer.org/wiki/Documentation/Nightly/Extensions/DTI_ALPS

2 https://github.com/LOAMRI/Slicer-DTI-ALPS
